# The Social Norms of Suicidal and Self-Harming Behaviours in Scottish Adolescents

**DOI:** 10.3390/ijerph14030307

**Published:** 2017-03-15

**Authors:** Jody Quigley, Susan Rasmussen, John McAlaney

**Affiliations:** 1 Division of Psychology, University of Stirling, Stirling FK9 4LA, UK; 2 Department of Psychological Sciences and Health, University of Strathclyde, Glasgow G1 1QE, UK; s.a.rasmussen@strath.ac.uk; 3 Psychology Research Group, Bournemouth University, Fern Barrow, Poole BH12 5BB, UK; jmcalaney@bournemouth.ac.uk

**Keywords:** suicide, self-harm, social norms, normative perception, social influence

## Abstract

Although the suicidal and self-harming behaviour of individuals is often associated with similar behaviours in people they know, little is known about the impact of perceived social norms on those behaviours. In a range of other behavioural domains (e.g., alcohol consumption, smoking, eating behaviours) perceived social norms have been found to strongly predict individuals’ engagement in those behaviours, although discrepancies often exist between perceived and reported norms. Interventions which align perceived norms more closely with reported norms have been effective in reducing damaging behaviours. The current study aimed to explore whether the Social Norms Approach is applicable to suicidal and self-harming behaviours in adolescents. Participants were 456 pupils from five Scottish high-schools (53% female, mean age = 14.98 years), who completed anonymous, cross-sectional surveys examining reported and perceived norms around suicidal and self-harming behaviour. Friedman’s ANOVA with post-hoc Wilcoxen signed-ranks tests indicated that proximal groups were perceived as less likely to engage in or be permissive of suicidal and self-harming behaviours than participants’ reported themselves, whilst distal groups tended towards being perceived as more likely to do so. Binary logistic regression analyses identified a number of perceived norms associated with reported norms, with close friends’ norms positively associated with all outcome variables. The Social Norms Approach may be applicable to suicidal and self-harming behaviour, but associations between perceived and reported norms and predictors of reported norms differ to those found in other behavioural domains. Theoretical and practical implications of the findings are considered.

## 1. Introduction

A number of social factors have been identified which may impact upon individuals’ risk of engaging in suicidal and self-harming behaviour (SSHB hereafter for brevity), including social support and connectedness [1], socioeconomic deprivation [2], and media reporting of suicide [3,4]. Clustering of SSHB has been repeatedly observed in young people [5,6,7], and in those with mental health issues [6,7,8], suggesting these groups may be particularly susceptible to a contagion-like spread of SSHB. Self-harming behaviours are more prevalent in young people than in older groups [9,10], reportedly occurring in children as young as 5 [11] with a peak between 14 and 18 years [12]. Given that self-harm represents a major risk factor for future suicide [13,14,15], young people represent a particularly high-risk group for SSHB, and social factors contributing to that risk may be especially significant within this group.

A systematic review of the literature suggests that the SSHB of family, friends, peers and others in children’s and adolescents’ social networks is strongly associated with their own risk of engaging in SSHB [16]. However, research has tended to assume individuals’ accurate knowledge of the behaviour of others, often based on factors such as attendance at the same school or membership of the same family. The potential for inaccuracy of perceptions or the impact of more general beliefs about others’ typical engagement in SSHB has generally not been considered.

Perceived social norms surrounding a given behaviour are evidenced to influence an individual’s own engagement in and permissiveness towards that behaviour. Both the normative rates of engagement in a particular behaviour (descriptive norms) and the normative attitude or level of permissiveness towards a given behaviour (injunctive norms) are associated with self-reported behaviour and attitudes. Despite this, individuals tend to believe that others behave in more damaging or negative ways than reported norms would suggest [17,18,19]. Perceptions of group norms surrounding a particular behaviour consistently show strong positive associations with self-reports of engagement in and permissiveness towards that behaviour, with those with higher reported norms perceiving norms to be particularly high [20]. These effects have been identified in a broad a range of behavioural domains, including alcohol consumption [17,18,19], gambling [21], risky sex [22], seatbelt use [23], substance use [24], and snacking behaviour [25]. A relatively small number of studies have found no direct relationship between perceived norms and individuals’ own behaviour, but where this is the case, samples may be small and the reference groups unspecific and distal [26], or norms do predict behaviour but only under particular conditions [27]. There have been wider criticisms of the methodological basis of both social norms research and the Social Norms Approach [28], although these criticisms have themselves been challenged [29]. A recent systematic review of the Social Norms Approach concluded that there may be some issues with how social norms campaigns are implemented, but that there is sufficient evidence to support the central premise of the approach that people do overestimate harmful behaviours and attitudes in their peers [30].

Interventions have been developed using the Social Norms Approachin a number of behavioural domains. Social norms surveys are used to measure a target group’s reported behaviour and attitudes (reported norms), and their perceptions of the average behaviour and attitudes of others (perceived norms), and where perceived norms differ (in a negative/unhealthy direction) from reported, reported norms are fed back to the target group, with the aim of aligning perceived norms more closely with reported norms; thereby reducing damaging behaviours or increasing positive ones. The Social Norms Approach has effectively reduced a number of damaging behaviours/attitudes, including alcohol consumption [31], substance use [32], drink-driving [33], bullying [34], and rape-supportive attitudes [35], and increased a number of positive behaviours/attitudes, such as the reporting of bullying [34], sun-protection behaviours [36], HIV-prevention behaviours [37], environmental conservation behaviours [38], and positive sexual attitudes [39]. Again, whilst social norms interventions can be very effective in changing behaviour, a small number of campaigns have failed to show any effect of altering perceptions on subsequent behaviour [40].

The current study aimed to extend the literature around social norms to include SSHB. In addition to associations consistently found between SSHB and many of the behaviours to which the Social Norms Approach has already been applied (including alcohol consumption and substance use; e.g., [41,42]), previous work by the current authors (submitted manuscript) suggests that undergraduate students’ SSHB is predicted by their normative perceptions of SSHB. Given that research has identified relationships between children and adolescents’ own SSBH and that of people they know [16], it was considered likely that adolescents’ engagement in SSHB may also be related to their normative perceptions of those behaviours—regardless of the accuracy of those perceptions. Further, young people are particularly susceptible to influence from their social environment [43,44], and evidence suggests that those who are most prone to influence may be at an already heightened risk of engaging in dangerous or risky behaviours [45]. If heightened perceptions of social norms around SSHB are positively associated with adolescents’ own engagement in SSHB, this could have dangerous implications in terms of increasing their risk of harm. The current study therefore aimed to determine whether the Social Norms Approach might be applicable to SSHB in adolescents, through identifying whether discrepancies exist between perceived and reported norms, and whether perceived norms predict adolescents’ reported norms. It was hypothesised that discrepancies would be observed between perceived and reported norms around SSHB, and that perceptions of normative behaviour and attitudes around SSHB would be associated with individuals’ own reported behaviour and attitudes. Should this be the case, it might provide professionals working with young people (e.g., in schools or other socially-oriented settings) with opportunities for intervention and prevention, via the Social Norms Approach.

## 2. Materials and Methods

### 2.1. Participants

Participants were 456 pupils from five mainstream Scottish high-schools, spanning four different Local Education Authorities (LEAs). Two schools were situated in large urban areas and three in other urban areas (based on the Scottish Government 6 Fold Urban Rural Classification), and they ranged from 5 to 10 in Scottish Index of Multiple Deprivation deciles (where 1 = most deprived, 10 = least deprived). 52.63% of participants were female, with ages ranging 11–17 years (mean = 14.98, SD = 1.09). One participant did not report their gender, and two did not report their age. More detailed breakdown of these characteristics can be found in Supplemental Table S1.

### 2.2. Measures

The self-report survey instrument was developed through consultation of the literature and previous social norms surveys [21,24]. Surveys were paper-based and responses were obtained using a multiple choice, tick-box-style format. As with previous social norms research, questions were tailored specifically to suit the population and the behaviours under investigation. Participants were asked about their thoughts of self-harm, acts of self-harm, thoughts of suicide, and suicide attempts (descriptive norms), and their permissiveness towards self-harm and suicide attempts (injunctive norms). Self-harm was defined as “deliberately taking an overdose (e.g., pills or other medication), or trying to harm yourself in some other way (such as cutting yourself)”, as per the CASE study [46]. Suicidal ideation was defined as “thinking about attempting to end your life (i.e., deliberately die by suicide)”, and suicide attempt was defined as “attempting to end your life (i.e., deliberately die by suicide)”. Their responses to these items represented the *reported norms*. Each reported norm question was paired with a question examining participants’ normative perceptions of that same behaviour or attitude in their close friends, their parents, their extended family, high-school pupils the same age and sex as them, pupils at their high-school, high-school pupils in general, people their age in general and people in general. Their responses to these questions represented the *perceived norms*.

Descriptive norms: Self-reported descriptive norms questions were posed in the format “*Have you ever (thought about harming yourself/harmed yourself/thought about ending your life/attempted to end your life)?*”, and matching items regarding participants’ perceptions of norms were posed in the format “*Do you think the following people ever (think about harming themselves/harm themselves/think about ending their lives/attempt to end their lives)?*”. Responses to both were given on a 5-point scale: “*never*”, “*have done occasionally in the past*”, “*have done regularly in the past*”, “*do so occasionally*” and “*do so regularly*”.

Injunctive norms: Self-reported injunctive norms questions were posed in the format “*Which statement about (harming oneself/attempting to end one’s life) do you feel best represents your own attitude?*”, and matching normative perception questions were posed in the format “*Which statement about (harming oneself/attempting to end one’s life) do you feel best represents the attitudes of the following people?*”. Responses to each were given on a 3-point scale: “*completely wrong*”, “*understandable under certain circumstances*” or “*completely OK*”. These SSHB questions were embedded within the context of a larger social norms study investigating risky health behaviours in general (including such behaviours as smoking, seatbelt use and help-seeking), the findings of which are reported elsewhere. This larger study also had the advantage of avoiding the over-emphasis of SSHB in a vulnerable population.

### 2.3. Procedure

Full ethical approval for the study was obtained from the University of Strathclyde Ethics Committee (project ID code: UEC13/26; approval date: 5 June 2013). All 32 LEAs in Scotland were contacted in the first instance and provided with study information and permission was requested to contact schools directly. Head-teachers were then contacted, and arrangements for collection of parental assent (for those under 16 years of age) were made with the schools. Those under 16 s for whom parental assent was obtained, and over 16 s (for whom parental assent was not required by the institutional ethics committee), were then invited to participate. All pupils were given full study information and asked to provide written informed consent. They were then invited to complete the survey privately and anonymously, in a classroom setting during an ordinary lesson. Participation took 20–40 min, and surveys were collected in sealed envelopes by the researcher.

### 2.4. Analyses

Reported norms (self-reports of adolescents’ own behaviours and attitudes) were compared with perceived norms (perceptions of others’ behaviour and attitudes) for each of the reference groups to determine if there were significant differences between the behaviour/attitude of respondents and what they perceived to be the norm. In keeping with the terminology used in previous social norms research, this refers to a self-other discrepancy. Given that data was ordinal and had no clear numerical interpretation, and that SSHB does not tend to be normally distributed, non-parametric analyses were performed. Friedman’s ANOVA was selected for this analysis as it detects differences between groups across multiple tests (using analysis of the variance of ranks) where data is ordinal. In order to determine where any differences were found, post-hoc Wilcoxen signed-ranks tests (with Bonferonni corrections) were used.

Whether or not there were any associations between perceived and reported norms was also examined. Due to the relative rarity with which some behaviours were reported (e.g., “I attempt suicide regularly/often”), descriptive norms responses were re-coded into a binary variable denoting ever having engaged in a given behaviour (1) and never having engaged in that behaviour (0), and injunctive norms responses were recoded into believing a behaviour to be completely wrong (0), and believing it be OK/understandable at least in some circumstances (1). Binary logistic regression was then used to determine predictors of reported norms, odds ratios and 95% confidence intervals. Separate regressions were run for each of the four descriptive norms and the two injunctive norms. For all self-harm-related outcome variables, predictors entered into the regression were age, sex, perceptions of all others’ thoughts of self-harm, self-harm and permissiveness of self-harm. For all suicide-related outcome variables, age, sex, perceptions of all others’ thoughts of suicide, suicide attempts, and permissiveness of suicide were entered into the regression. Collinearity diagnostics revealed some multicollinearity in three of the models, with tolerance levels <0.1 and VIF >10 for models 1, 2, and 4 (thoughts of self-harm, self-harm and permissiveness of self-harm). It is advised that multicollinearity should be acknowledged and potential bias considered, but all variables should be maintained in the model in order to avoid further complications associated with removing them [47]. All variables were therefore preserved in their respective models.

## 3. Results

Table 1 illustrates the reported norms of the sample. Figures refer to the number of participants reporting ever having engaged in a particular behaviour, or believing that a particular behaviour is ever “OK”.

All six Friedman’s ANOVAs indicated differences between reported and perceived norms. Table 2 presents the results, with significantly different reference groups identified through post-hoc Wilcoxen signed-ranks tests. Full results (including non-significant differences) can be found in Supplemental Table S2.

Results of the six binary logistic regressions (Models 1–6) are presented in Table 3, with model statistics. One model estimates predictors of each reported norm. Only variables identified as significantly associated with outcome variables are reported, but full results with all model variables can be found in Supplemental Table S3.

### 3.1. Thoughts of Self-Harm

Reported thoughts of self-harm were significantly different to perceived thoughts of self-harm. Post-hoc tests indicated parents and extended families were perceived as less likely to have thoughts of self-harm than participants reported, whilst all other groups were perceived as more likely. 27.0%–44.8% of the variance in reported thoughts of self-harm was explained by Model 1. Those who believed their friends had thoughts of self-harm or engaged in self-harm were around one and a half times more likely to report having thoughts of self-harm themselves, whilst this increased to almost six times more likely for perceptions that family members engaged in self-harm. The belief that pupils from the same school have thoughts of self-harm was associated with a decrease by approximately half in reported thoughts of self-harm. Inspection of collinearity diagnostics indicated that there may be some multicollinearity, so the model should be interpreted with caution.

### 3.2. Self-Harm

For self-harm, reported norms also differed from perceived norms. Parents and extended families were perceived as less likely to engage in self-harm than participants reported. All other groups were perceived as more likely. Model 2 accounted for 24.2%–46.8% of the variance in reported self-harm. Females were almost four times more likely than males to report engaging in self-harm. Those who believed their friends had thoughts of self-harm or that high-school pupils the same sex as them had thoughts of self-harm were around twice as likely to report self-harming, but believing that pupils at the same school had thoughts of self-harm was negatively associated with reported self-harm. As the variables in the current model are identical to those in the previous, multicollinearity was again indicated.

### 3.3. Thoughts of Suicide

Reported thoughts of suicide significantly differed from perceptions of others’ thoughts of suicide. Parents and extended families were perceived as less likely to have thoughts of suicide than participants’ own reported thoughts. High-school pupils of the same age and sex, high-school pupils attending the same school, high-school pupils in general, and people in general were all perceived as more likely. 19.4%–37.8% of the variance in reported thoughts of self-harm was explained by Model 3. Those who believed their close friends had thoughts of suicide were over three times more likely to report thoughts of suicide, whilst believing friends had made suicide attempts was associated with a decrease in own thoughts of suicide.

### 3.4. Suicide Attempts

Significant differences were again found between reported and perceived norms for suicide attempts. High-school pupils of the same age and sex, high-school pupils attending the same school, high-school pupils in general, and people in general were all perceived as more likely than reported norms, to make suicide attempts. The only significant predictor in Model 4 was perceptions of close friends’ permissiveness towards suicide attempts, which was associated with an almost thirty times increased likelihood of reporting suicide attempts, but the overall model was not significant.

### 3.5. Permissiveness of Self-Harm

Reported norms for permissiveness of self-harm differed significantly from perceptions of permissiveness of self-harm. Close friends, parents and extended families were all perceived as less likely to be permissive of self-harm than reported norms. Model 5 explained 38.3%–51.2% of the variance in reported permissiveness. Females were more than twice as likely as males to report permissive attitudes. Believing one’s friends hold permissive attitudes was associated with a more than four times increased likelihood of reporting permissive attitudes, and those who believed people in general held permissive attitudes were more than three times more likely to report permissive attitudes. Inspection of collinearity diagnostics indicated that again there may be some multicollinearity between variables, so the model should be interpreted with caution.

### 3.6. Permissiveness of Suicide Attempts

Finally, it was also found that significant differences existed between reported and perceived permissiveness of suicide attempts. Close friends, parents and extended families were all perceived as less likely to be permissive of suicide attempts than participants reported themselves. 46.5%–62.5% of the variance in reported permissiveness was accounted for by Model 6. Females were about three times as likely as males to report permissive attitudes. Those who believed pupils the same age and sex as them had thoughts of suicide were around one and a half times more likely to hold permissive attitudes, while this increased to just over six times for those who believed their friends held permissive attitudes, and almost thirty times for those who believed their family held permissive attitudes.

## 4. Discussion

The aim of the current study was to explore the social norms of SSHB in adolescents and to examine whether discrepancies exist between perceived and reported norms for SSHB. The study also aimed to identify whether adolescents’ own SSHB could be predicted by their normative perceptions of a number of reference groups. Significant self-other discrepancies were indeed observed for all four behavioural outcome variables (thoughts of self-harm, self-harm, thoughts of suicide and suicide attempts) and for both attitudinal outcome variables (permissiveness towards self-harm and towards suicide attempts) but discrepancies were only significant for certain reference groups and were not always in the predicted direction.

Approximately 10% of males, and almost 25% of females reported having had thoughts about self-harm, which is comparable to previous European studies [46]. Similarly, our 4% of males reporting having engaged in self-harm is in line with previous studies, and the 20% of females in our sample reporting having engaged in self-harm is comparable to the 17% reported in England and Australia in the CASE study [46] (though it is somewhat higher than rates reported in other Scottish studies, such as [48]). Our sample reported rates of suicidal ideation and attempts at approximately 13% and 4% (respectively), which are also comparable to previous studies in Europe and the US [49,50].

### 4.1. Descriptive Norms

There was an overall tendency for participants to believe that proximal groups were less likely, and distal groups more likely, to engage in SSHB than self-reported norms. Parents and family members were perceived as less likely than reported norms to think about self-harm, engage in self-harm, or think about suicide, whilst more distal groups were perceived as more likely to do so. Interestingly, close friends were perceived more similarly to distal groups; with participants reporting that close friends were more likely to engage in SSHB than reported norms.

That proximal groups were perceived as less likely to engage in SSHB than reported norms is contrary to previous social norms research, but perceived norms for distal groups followed a similar pattern to that generally observed in social norms research (i.e., that others are perceived as more likely than oneself to engage in negative or damaging behaviours). It is possible that adolescents simply have access to more accurate knowledge about the behaviour of those close to them, and that having never seen any evidence of SSHB amongst their friends and family, they correctly surmise that they do not engage in SSHB. It has previously been shown that self-other discrepancies are smaller for proximal groups than for distal groups, for this reason [51]. However, as previous social norms studies have still found discrepancies for proximal groups (albeit smaller ones), this explanation may not be sufficient. An alternative is that our differing findings are indicative of inherent differences in the way that SSHB is perceived in comparison with previously studied behaviours (e.g., alcohol consumption) in terms of rightness/wrongness, positivity/negativity, personal responsibility, and their impact on others. For example, links with psychological distress and mental ill-health may mean that as adolescents do not believe many of their loved ones to be mentally unwell, SSHB is perceived as unlikely to occur in proximal groups. They know that mental illness *does* exist, so it is perceived as something that must occur elsewhere (i.e., in more distal groups) by default. Similarly, adolescents may believe that due to the impact that others’ SSHB would have on themselves (e.g., distress caused by a loved one suffering) they would be aware if those close to them had harmed themselves or attempted suicide, whereas they perhaps would not be so personally affected if loved ones engaged in more traditionally studied behaviours (such as alcohol consumption, for example). As they have not been impacted in this way, they may assume it must not have happened. These potential effects of perceptions around SSHB in comparison to other behaviours would suggest that in order to interpret the findings of *any* social norms research appropriately, a better understanding is required of the ways in which participants perceive and understand the behaviour of interest. The discrepancy in the way that different groups were perceived in the current study also argues for the inclusion of a range of diverse reference groups in future social norms research.

Suicide attempts were perceived slightly differently to the other behaviours, with all groups (except parents) perceived as more likely to make suicide attempts than reported norms—although these discrepancies were only significant for distal group norms. Whilst previous social norms research has examined a number of risky and damaging behaviours, the ultimate aim of those behaviours tends not to be inflicting harm/death, as it (usually) is with SSHB, so it is perhaps unsurprising that our findings in this regard were so different to those of previous studies. It is interesting that parents were perceived as different to all other groups and uniquely immune to SSHB. This may be accounted for by the fact that the number of parents any individual has tends to be a relatively small number in comparison to larger reference groups (e.g., extended family, people your age), necessarily reducing the likelihood of any behaviour occurring within that limited group. Alternatively, it may represent a form of optimism bias, whereby the thought of a parent being hurt represents the kind of negative event that individuals believe is unlikely to happen to them [52]. Differential effects on an individual of others dying by suicide, compared to their engagement in other SSHBs, has been noted elsewhere [16].

### 4.2. Injunctive Norms

For both attitudinal outcome variables (permissiveness of self-harm and of suicide attempts), there were significant discrepancies between perceived and reported norms, as predicted. However, again these discrepancies were not in the expected direction, with proximal groups perceived as less likely than reported norms to be permissive of both self-harm and suicide attempts. Distal groups tended to be perceived as more permissive of self-harm than reported norms—consistent with previous social norms literature—but not significantly. As previously mentioned, findings may demonstrate adolescents’ enhanced knowledge of the attitudes of those close to them, in comparison to more distal, unfamiliar groups. However, the differences in the direction of discrepancies between the current findings and previous social norms findings suggest that SSHB is perceived somewhat differently to behaviours studied previously, perhaps in terms of their ethical or moral status, their links with mental illness, and their effect on others (particularly in the case of suicide).

The finding that distal groups tended towards being perceived as more likely to be permissive of self-harm may be indicative of a belief that permissiveness is a negative feature, reflecting similar results to those found in previous social norms research (i.e., individuals believe that others behave in worse ways than they do themselves). Evidence suggests that whilst attitudes towards SSHB vary across individuals [53,54], many people hold very negative and “blaming” views [55,56,57,58], which may shape their perceptions of others’ attitudes. The distinction between proximal and distal groups in this regard may reflect in-group/out-group biases [59,60], with those deemed part of participants’ in-group (e.g., friends, family) perceived as behaving differently (better) to those in out-groups (e.g., people not known to the individual).

### 4.3. Predictors of Reported Norms

Significant predictor models were generated for five out of the six outcome variables (thoughts of self-harm, self-harm, thoughts of suicide, permissiveness of self-harm and permissiveness of suicide attempts), and each model had a number of significant predictors. The models accounted for a substantial proportion of the variance in the independent variables (e.g., as much as 62.5% in the case of permissiveness of suicide attempts). Again, associations with perceived norms were not always in the predicted direction, based on previous social norms research (i.e., that greater/more permissive perceived norms will predict greater/more permissive reported norms). As already described, gender predicted some of the outcome variables, but age was not associated with any, and descriptive norms tended to more often predict self-reported norms than did injunctive norms. Generally speaking, perceived proximal group norms were more often positively than negatively associated with reported norms, whilst distal group norms were roughly as equally likely to be positively associated with reported norms as negatively. Aside from these features, there was no clear, discernible pattern in the variables associated with outcomes.

One clear pattern that did emerge was that the perceived norms of close friends were particularly important in predicting reported norms, with at least one close friends-related norm associated with each of the six outcome variables. The wide-reaching impact of peers on self-harming behaviour in adolescents has been well-documented [61], and our findings appear to support this; suggesting that perceived norms pertaining to close friends may be particularly influential in increasing adolescents’ engagement in and permissiveness towards SSHB. Stronger associations between reported norms and the perceived norms of proximal groups (relative to distal groups) have been shown in previous social norms research [62,63]. However, the need for peer approval during adolescence has been highlighted previously [64], as have socialisation and modelling effects [6], so the mechanisms of peer influence over SSHB may be multiple [61]. A review of peer influences on alcohol consumption [17] identified 3 specific processes through which influence occurs; overt encouragement, modelling, and perceived social norms. The latter 2 in particular may be relevant in this context; although it is not impossible that all 3 play a part in the case of close friendships.

The finding that perceived descriptive norms more often predicted reported norms than did injunctive norms (with a ratio of 2:1) is contrary to patterns shown in previous social norms research, which generally finds injunctive norms to be better predictors of reported norms [18]. There are several possible reasons for this. Firstly, given that a number of relationships increase in intimacy and perceived significance as a function of age [65,66], the mutual holding of shared values deemed important in the maintenance of successful relationships in older individuals [67,68] may be considered less so in adolescents, such that the (perceived) beliefs and attitudes of those around them are less influential in shaping their own. Secondly, given that adolescents may be particularly prone to egocentrism [69], making inferences about others’ attitudes and beliefs may simply not be of interest to them, whilst actual behaviours are more visible and salient, and require less outward-focused thought. Finally, a relative lack of knowledge of other people on account of adolescents’ age and inexperience may render them unfamiliar with other people’s thoughts and attitudes, such that they are less likely to use them as a source of information or guidance. Despite this however, some previous research exists which supports the current findings that descriptive norms are better predictors of reported norms than are injunctive norms [62].

### 4.4. Implications

The current findings suggest that as found in previous social norms research, there were discrepancies between perceived and reported norms for adolescent SSHB, and certain perceived norms predicted individuals’ own behaviour and attitudes. As such, social norms interventions which feed back normative information relating to SSHB in order to align perceived norms more closely with reported norms, thereby reducing any related increase in individuals own behaviour, may be applicable within this population. As the impact of perceived on reported norms differs slightly from previous social norms research in the current study, any intervention would need to be carefully designed, taking into consideration the specific behaviour or attitude in question, and the nature of the reference groups used. As descriptive norms appeared particularly salient to this age group, an appropriate social norms intervention might be one which feeds back information regarding the relatively low reported incidence of SSHB in this sample, framing messages in terms of “people your age/sex” or “people in general”. As friends’ norms also appeared particularly salient, these messages could be shaped to infer that their friends constitute part of this group, and therefore have similarly low rates of engagement in SSHB.

More generally, the observation that certain perceived norms—particularly those of friends—were positively associated with individuals’ own reported behaviour and attitudes, has important practical implications for families and professionals caring for or working with adolescents, and suggests that perception may play a significant part in eliciting an imitative or contagion-like spread of SSHB. It is important that measures are taken to ensure that the social environments in which adolescents function are healthy, supportive and open, and that efforts are made to prevent the development of a culture in which SSHB is perceived as ubiquitous or normalised. It is also vital that young people are provided with psychoeducation on issues around psychological distress, alternative coping strategies, and the importance of seeking support.

One vital consideration when designing interventions based on the Social Norms Approach—or indeed social influence more generally—is the importance of striking a balance between the avoidance of “normalising” particular behaviours, and the exacerbation of stigma and/or feelings of isolation in those who engage in them. This is particularly important with regard to SSHB, given that engagement therein may be increased by feelings of social isolation [70,71,72,73] and experiences of stigma [74,75]. The thoughtful and sensitive design of interventions conveying supportive, non-judgemental messages is thus imperative in order to avoid inadvertent increases in harm. That social norms interventions are not always effective in changing behaviour should also be considered [26,40], and additional support and preventative measures should continue to be employed to help protect young people from harm.

### 4.5. Limitations

The study is subject to some limitations, including challenges associated with appropriate wording of the novel survey instrument, and issues around defining meaningful reference groups (perhaps accounting for some of the multicollinearity observed). The survey was not piloted in an adolescent sample (only on undergraduate students sampled in the authors’ previous work), so there may have been issues around comprehensibility and relevance to the sample, although attempts were made to adapt the wording and reference groups for increased age-appropriateness. Despite efforts to recruit as widely as possible, only five schools agreed to participate, and although they were relatively varied in their socioeconomic characteristics, they were all from urban or semi-urban settings, so representativeness cannot be guaranteed. Finally, there are limits to the inferences that can be made from the current findings given that the data was cross-sectional (so assumptions about the causal direction of effects cannot be confirmed) and that some potential multicollinearity was indicated for some of the models. Future research should aim to replicate the current findings using larger samples (particularly including schools in rural settings), longitudinal methods (in order to address causality), and carefully defined reference groups.

## 5. Conclusions

The current findings differed quite substantially from those obtained in previous social norms research. Discrepancies between perceived and reported norms appear consistent, but different reference groups appear to be perceived differently and vary in their predictive power over reported norms. Although cross-sectional in design (and therefore unable to confirm causal direction), a number of perceived social norms appear predictive of adolescents’ behaviour and attitudes in this domain, and the regression models generated appear to account for substantial proportions of the variance in reported norms. The fact that perceived norms are at all predictive of reported norms argues for the relevance of the Social Norms Approach within the domain of SSHB, and the potential for the development of interventions based on the approach for the reduction of SSHB. Descriptive norms appear to be particularly important to this population, such that interventions which aim to utilise normative information to reduce SSHB in this group may do well to employ a particular focus on behaviour, relative to attitudes. A number of issues remain unclear, and further research is required to help explain in more detail some of the unexpected findings of the current study, and to continue to explore whether and how the Social Norms Approach might be applicable to the reduction of SSHB in young people.

## Figures and Tables

**Table 1:** Descriptive data relating to reported norms.

Sex	Descriptive Norms				Injunctive Norms	
	Thoughts of Self-Harm *N* (%)	Thoughts of Suicide *N* (%)	Self-Harm *N* (%)	Suicide Attempts *N* (%)	Permissiveness of Self-Harm *N* (%)	Permissiveness of Suicide Attempts *N* (%)
Males	21 (9.72)	9 (4.17)	20 (9.30)	7 (3.26)	67 (31.16)	61 (28.64)
Females	59 (24.89)	48 (20.34)	37 (15.68)	11 (4.68)	145 (62.23)	130 (56.28)
Total	80 (17.66)	57 (12.61)	57 (12.64)	18 (4.00)	212 (47.32)	191 (43.02)

**Table 2:** Results of Friedman’s ANOVA with post-hoc Wilcoxen signed-ranks to determine difference between perceived and reported norms.

	Reported Norm Friedman’s ANOVA	Reference Group	Wilcoxen Signed-Ranks Tests			
			Direction of Diff. ^4,5^	T Statistic	*p*-Value	Effect Size
**Descriptive Norms**	Thoughts of SH ^1^ χ^2^ (7) = 1402.04, *p* < 0.001	Friends	S < O	2003.00	0.005	0.09
		Parents	S > O	225.00	<0.001	0.23
		Family	S > O	686.00	<0.001	0.18
		Pupils the same sex/age	S < O	1105.50	<0.001	0.46
		Pupils at same school	S < O	1511.00	<0.001	0.49
		Pupils in general	S < O	1249.00	<0.001	0.50
		People in general	S < O	1293.00	<0.001	0.49
	SH χ^2^ (7) = 1440.99, *p* < 0.001	Friends	S < O	1207.00	<0.001	0.12
		Parents	S > O	153.50	<0.001	0.20
		Family	S > O	541.00	<0.001	0.13
		Pupils the same sex/age	S < O	817.50	<0.001	0.47
		Pupils at same school	S < O	672.00	<0.001	0.51
		Pupils in general	S < O	656.00	<0.001	0.52
		People in general	S < O	444.00	<0.001	0.52
	Thoughts of suicide χ^2^ (7) = 1270.49, *p* < 0.001	Parents	S > O	274.50	<0.001	0.16
		Family	S > O	595.50	<0.001	0.10
		Pupils the same sex/age	S < O	950.50	<0.001	0.41
		Pupils at same school	S < O	1233.50	<0.001	0.44
		Pupils in general	S < O	1193.00	<0.001	0.48
		People in general	S < O	1131.50	<0.001	0.50
	SA ^2^ χ^2^ (7) = 1184.28, *p* < 0.001	Pupils the same sex/age	S < O	213.00	<0.001	0.41
		Pupils at same school	S < O	234.00	<0.001	0.42
		Pupils in general	S < O	113.50	<0.001	0.50
		People in general	S < O	80.50	<0.001	0.53
**Injunctive Norms**	Perm ^3^ of SH χ^2^ (7) = 275.14, *p* < 0.001	Friends	S > O	1097.50	<0.001	0.15
		Parents	S > O	984.00	<0.001	0.28
		Extended family	S > O	1084.00	<0.001	0.23
	Perm of SA χ^2^ (7) = 190.85, *p* < 0.001	Friends	S > O	702.00	<0.001	0.18
		Parents	S > O	595.00	<0.001	0.24
		Extended family	S > O	450.00	<0.001	0.23

^1^ SH = self-harm; ^2^ SA = suicide attempt; ^3^ Perm = permissiveness (injunctive norms); ^4^ S = self (reported norm); ^5^ O = other (perceived norm).

**Table 3:** Binary logistic regression analyses of variables significantly associated with reported norms.

Own reported Behavior with Model Statistics	Significant Predictor Variables	*p*-Value	Odds Ratio (OR)	95% Confidence Interval for OR	
				Lower	Upper
*Model 1: Thoughts of SH* ^1^ χ^2^ (23) = 103.37, *p* < 0.001 R^2^ = 0.27 (Cox & Snell), 0.45 (Nagelkerke)	Friends thoughts of SH	0.044	1.492	1.011	2.201
	Friends SH	0.043	1.495	1.013	2.205
	Family SH	0.028	5.818	1.215	27.873
	Pupils at same school thoughts of SH	0.028	0.509	0.279	0.929
*Model 2: SH* χ^2^ (23) = 90.94, *p* < 0.001 R^2^ = 0.24 (Cox & Snell), 0.47 (Nagelkerke)	Sex (male)	0.017	0.229	0.068	0.770
	Friends thoughts of SH	0.017	1.760	1.106	2.800
	Pupils the same age/sex thoughts of SH	0.034	2.252	1.063	4.770
	Pupils at same school thoughts of SH	0.045	0.504	0.258	0.9783
*Model 3: Thoughts of suicide* χ^2^ (23) = 70.33, *p* < 0.001. R^2^ = 0.19 (Cox & Snell), 0.38 (Nagelkerke)	Friends thoughts of suicide	< 0.001	3.388	1.926	5.959
	Friends SA	0.006	0.312	0.136	0.717
*Model 4: SA* ^2^ χ^2^ (23) = 26.45, *p* = 0.280 R^2^ = 0.08. (Cox & Snell), 0.33 (Nagelkerke)	Friends perm of SA	0.029	29.858	1.410	632.265
*Model 5: Perm* ^3^ *of SH* χ^2^ (23) = 77.03, *p* < 0.001 R^2^ = 0.38 (Cox & Snell), 0.53 (Nagelkerke)	Sex (male)	0.010	0.428	0.225	0.814
	Friends perm SH	0.003	4.363	1.650	11.540
	People in general perm of SH	0.011	3.494	1.336	9.138
*Model 6: Perm of SA* χ^2^ (23) = 203.71, *p* < 0.001 R^2^ = 0.47 (Cox & Snell), 0.63 (Nagelkerke)	Sex (male)	0.004	0.345	0.169	0.706
	Pupils the same age/sex thoughts of suicide	0.037	1.590	1.029	2.458
	Friends perm of SA	0.003	6.208	1.853	20.797
	Family perm of SA	0.001	29.308	3.895	220.554

^1^ SH = self-harm; ^2^ SA = suicide attempt; ^3^ Perm = permissiveness (injunctive norms).

## Supplementary Materials

The following are available online at www.mdpi.com/1660-4601/14/3/307/s1, Table S1: Characteristics of schools and participants from each school; Table S2: Full results of Friedman’s ANOVA with post-hoc Wilcoxen signed-ranks to determine difference between perceived and reported norms; Table S3: Binary logistic regression analyses of all variables tested for associations with reported norms.
