# Supplementary material for: The Social Norms of Suicidal and Self-Harming Behaviours in Scottish Adolescents
Source: Int J Environ Res Public Health. 2017 Mar 15;14(3):307. doi: 10.3390/ijerph14030307 (PMC5369143; doi:10.3390/ijerph14030307)
Supplement: Supplementary file 1 [file ijerph-14-00307-s001.pdf]

Supplementary Materials: The Social Norms of Suicidal and Self-Harming Behaviours in Scottish Adolescents

Jody Quigley, Susan Rasmussen and John McAlaney

Table S1. Characteristics of schools and participants from each school.

**School [SIMD decile]* (urban/rural)****	**Gender**	**Age**								**TOTAL (% of whole sample)**
		**11**	**12**	**13**	**14**	**15**	**16**	**17**	**Missing**	
1	Males			5	32	12	15			64
[10]	Females			10	22	7	26		1	66
(other urban)	Total			15	54	19	41			130 (28.5)
2	Males			5	25					30
[8]	Females			4	25					29
(other urban)	Total			9	50					59 (12.9)
3	Males				8	1				9
[6]	Females				32	2				34
(other urban)	Total				40	3				43 (9.4)
4	Males					9	30			39
[9]	Females					7	43	1		51
(large urban)	Total					16	73	1		90 (19.7)
5	Males		4	25	24	21				75
[5]	Females	1	5	17	21	14				58
(large urban)	Missing				1				1	2
	Total	1	9	42	46	35			1	134 (29.4)
TOTAL		1	9	66	189	73	114	1	3	456

*SIMD decile: 1 = most deprived, 10 = least deprived. **Urban/rural classification based on Scottish Government 6 Fold Urban Rural Classification (large urban = population > 125,000; other urban = 10,000 – 124,999; accessible small town = 3,000 – 9,999 within 30 mins drive of settlement > 10,000; remote small town = 3,000 – 9,999 not within 30 mins of settlement > 10,000; accessible rural = < 3,000 within 30 mins drive of settlement > 10,000; remote rural = < 3,000 not within 30 mins drive of settlement > 10,000).

Table S2. Full results of Friedman's ANOVA with post-hoc Wilcoxen signed-ranks to determine difference between perceived and reported norms.

	Reported norm Friedman's ANOVA	Reference group	Wilcoxen signed-ranks tests: Direction of diff. [4, 5]	T statistic	P-value	Effect size
Descriptive norms	Thoughts of SH [1] $X^2(7) = 1402.04$, $p < .001$	Friends	S < O	2003.00	.005	.09
		Parents	S > O	225.00	<.001	.23
		Family	S > O	686.00	<.001	.18
		Pupils the same sex/age	S < O	1105.50	<.001	.46
		Pupils at same school	S < O	1511.00	<.001	.49
		Pupils in general	S < O	1249.00	<.001	.50
		People in general	S < O	1293.00	<.001	.49
	SH $X^2(7) = 1440.99$, $p < .001$	Friends	S < O	1207.00	<.001	.12
		Parents	S > O	153.50	<.001	.20
		Family	S > O	541.00	<.001	.13
		Pupils the same sex/age	S < O	817.50	<.001	.47
		Pupils at same school	S < O	672.00	<.001	.51
		Pupils in general	S < O	656.00	<.001	.52
		People in general	S < O	444.00	<.001	.52
	Thoughts of suicide $X^2(7) = 1270.49$, $p < .001$	Friends	S < O	1161.50	.943	.00
		Parents	S > O	274.50	<.001	.16
		Family	S > O	595.50	<.001	.10
		Pupils the same sex/age	S < O	950.50	<.001	.41
		Pupils at same school	S < O	1233.50	<.001	.44
		Pupils in general	S < O	1193.00	<.001	.48
		People in general	S < O	1131.50	<.001	.50
	SA [2] $X^2(7) = 1184.28$, $p < .001$	Friends	S < O	199.00	.018	.08
		Parents	S > O	48.50	.052	.07
		Family	S < O	172.50	.686	.01
		Pupils the same sex/age	S < O	213.00	<.001	.41
		Pupils at same school	S < O	234.00	<.001	.42
		Pupils in general	S < O	113.50	<.001	.50
		People in general	S < O	80.50	<.001	.53
Injunctive norms	Perm [3] of SH $X^2(7) = 275.14$, $p < .001$	Friends	S > O	1097.50	<.001	.15
		Parents	S > O	984.00	<.001	.28
		Family	S > O	1084.00	<.001	.23
		Pupils the same sex/age	S < O	2788.50	.595	.01
		Pupils at same school	S < O	2626.00	.150	.04
		Pupils in general	S < O	2875.50	.008	.01
		People in general	S < O	2662.00	.254	.08
	Perm of SA $X^2(7) = 190.85$, $p < .001$	Friends	S > O	702.00	<.001	.18
		Parents	S > O	595.00	<.001	.24
		Family	S > O	450.00	<.001	.23
		Pupils the same sex/age	S > O	1602.50	.070	.07
		Pupils at same school	S > O	1895.50	.113	.06

	Pupils in general	S > O	2520.50	.835	.01
	People in general	S < O	3211.00	.465	.03

[1] SH = self-harm, [2] SA = suicide attempt, [3] Perm = permissiveness (injunctive norms), [4] S = self (reported norm), [5] O = other (perceived norm).

Table S3. Binary logistic regression analyses of all variables tested for associated with reported norms.

Own reported behavior with model statistics	Predictor variables	P-value	Odds ratio (OR)	95% confidence interval for OR Lower	Upper
Model 1:	Sex (male)	.238	0.594	0.250	1.411
Thoughts of SH [1]	Age	.793	0.948	0.638	1.409
X^2 (23) = 103.37,	Friends thoughts of SH	.044	1.492	1.011	2.201
p < .001	Friends SH	.043	1.495	1.013	2.205
R^2 = .27 (Cox &	Parents thoughts of SH	.999	4.398E8	0.000	-
Snell), .45	Parents SH	1.00	1.437	0.000	-
(Nagelkerke)	Family thoughts of SH	.068	0.218	0.042	1.122
	Family SH	.028	5.818	1.215	27.873
	Pupils the same age/sex thoughts of SH	.088	1.681	0.926	3.050
	Pupils the same age/sex SH	.911	1.028	0.633	1.670
	Pupils at same school thoughts of SH	.028	0.509	0.279	0.929
	Pupils at the same school SH	.898	1.408	0.513	2.141
	High-school pupils in general thoughts of SH	.406	1.368	0.653	2.866
	High- school pupils in general SH	.744	0.884	0.423	1.849
	People in general thoughts of SH	.179	1.536	0.821	2.875
	People in general SH	.529	0.815	0.431	1.540
	Friends perm of SH	.363	1.634	0.568	4.704
	Parents perm of SH	.080	0.168	0.023	1.234
	Family perm of SH	.327	2.587	0.387	17.311
	Pupils the same age/sex perm of SH	.384	1.864	0.458	7.590
	Pupils at same school perm of SH	.899	1.112	0.217	5.694
	High-school pupils in general perm of SH	.550	0.639	0.148	2.771
	People in general perm of SH	.803	1.146	0.392	3.349
Model 2: SH	Sex (male)	.017	0.229	0.068	0.770
X^2 (23) = 90.94, p	Age	.405	1.247	0.742	2.095
< .001	Friends thoughts of SH	.017	1.760	1.106	2.800
R^2 = .24 (Cox &	Friends SH	.253	1.285	0.836	1.974
Snell), .47	Parents thoughts of SH	.000	8.782E8	0.000	-
(Nagelkerke)	Parents SH	1.00	1.490	0.000	-
	Family thoughts of SH	.358	0.623	0.227	1.708
	Family SH	.055	2.891	0.976	8.559
	Pupils the same age/sex thoughts of SH	.034	2.252	1.063	4.770
	Pupils the same age/sex SH	.212	0.694	0.391	1.231
	Pupils at same school thoughts of SH	.045	0.504	0.258	0.983
	Pupils at same school SH	.485	0.730	0.302	1.766
	High-school pupils in general thoughts of SH	.462	0.722	0.304	1.718
	High-school pupils in general SH	.200	1.777	0.737	4.287

	People in general thoughts of SH	.105	1.967	0.869	4.453
	People in general SH	.701	0.858	0.392	1.876
	Friends perm of SH	.268	2.031	0.580	7.115
	Parents perm of SH	.276	0.262	0.024	2.916
	Family perm of SH	.738	1.484	0.147	15.023
	Pupils the same age/sex perm of SH	.138	3.501	0.668	18.356
	Pupils at same school perm of SH	.649	0.633	0.088	4.538
	High-school pupils in general perm of SH	.524	0.571	0.102	3.195
	People in general perm of SH	.739	0.802	0.220	2.923
Model 3: Thoughts of suicide X^2 (23) = 70.33, p < .001. R^2 = .19 (Cox & Snell), .38 (Nagelkerke)	Sex (male)	.343	0.629	0.241	1.641
	Age	.096	0.693	0.450	1.068
	Friends thoughts of suicide	<.001	3.388	1.926	5.959
	Friends SA	.006	0.312	0.136	0.717
	Parents thoughts of suicide	.533	0.575	0.101	3.277
	Parent SA	.999	0.000	0.000	-
	Family thoughts of suicide	.861	0.922	0.369	2.299
	Family SA	.802	1.228	0.248	6.085
	Pupils the same age/sex thoughts of suicide	.066	1.650	0.967	2.816
	Pupils the same age/sex SA	.625	1.124	0.703	1.798
	Pupils at same school thoughts of suicide	.434	0.817	0.493	1.356
	Pupils at same school SA	.128	0.716	0.465	1.101
	High-school pupils in general thoughts of suicide	.622	0.848	0.442	1.630
	High-school pupils in general SA	.348	1.302	0.751	2.256
	People in general thoughts of suicide	.103	1.497	0.922	2.430
	People in general SA	.285	0.791	0.515	1.216
	Friends perm of SA	.313	2.121	0.492	9.146
	Parents perm of SA	.088	0.144	0.016	1.336
	Family perm of SA	.287	3.015	0.395	23.007
	Pupils the same age/sex perm of SA	.072	0.174	0.026	1.166
	Pupils at same school perm of SA	.125	6.618	0.592	73.990
	High-school pupils in general perm of SA	.473	0.435	0.045	4.227
	People in general perm of SA	.432	1.564	0.513	4.763
Model 4: SA [2] X^2 (23) = 26.45, p = .280 R^2 = .08. (Cox & Snell), .33 (Nagelkerke)	Sex (male)	.899	1.132	0.168	7.636
	Age	.617	0.827	0.394	1.738
	Friends thoughts of suicide	.677	0.799	0.278	2.297
	Friends SA	.363	1.840	0.495	6.836
	Parents thoughts of suicide	.402	2.522	0.290	21.958
	Parent SA	.999	0.000	0.000	-
	Family thoughts of suicide	.383	1.653	0.535	5.104
	Family SA	.848	0.717	0.024	21.654
	Pupils the same age/sex thoughts of suicide	.878	0.935	0.395	2.215
	Pupils the same age/sex SA	.731	0.840	0.312	2.262
	Pupils at same school thoughts of suicide	.542	1.354	0.511	3.590

	Pupils at same school SA	.601	1.272	0.516	3.133
	High-school pupils in general thoughts of suicide	.262	0.523	0.168	1.625
	High-school pupils in general SA	.520	0.702	0.238	2.065
	People in general thoughts of suicide	.330	1.504	0.662	3.416
	People in general SA	.413	1.389	0.633	3.047
	Friends perm of SA	.029	29.858	1.410	632.265
	Parents perm of SA	.511	0.245	0.004	16.308
	Family perm of SA	.882	0.726	0.011	49.939
	Pupils the same age/sex perm of SA	.128	0.040	0.001	2.529
	Pupils at same school perm of SA	.115	18.856	0.487	730.160
	High-school pupils in general perm of SA	.945	0.875	0.019	39.645
	People in general perm of SA	.754	0.699	0.074	6.569
Model 5: Perm [3] of SH X^2 (23) = 77.03, p < .001 R^2 = .38 (Cox & Snell), .53 (Nagelkerke)	Sex (male)	.010	0.428	0.225	0.814
	Age	.268	1.188	0.875	1.613
	Friends thoughts of SH	.850	1.038	0.704	1.532
	Friends SH	.334	1.242	0.801	1.925
	Parents thoughts of SH	.999	7.984E7	0.000	-
	Parents SH	1.000	1.175	0.000	-
	Family thoughts of SH	.083	0.337	0.099	1.151
	Family SH	.078	3.530	0.866	14.381
	Pupils the same age/sex thoughts of SH	.823	1.041	0.734	1.476
	Pupils the same age/sex SH	.705	1.069	0.758	1.506
	Pupils at same school thoughts of SH	.404	1.207	0.776	1.878
	Pupils at same school SH	.800	1.062	0.668	1.686
	High-school pupils in general thoughts of SH	.592	0.881	0.554	1.401
	High-school pupils in general SH	.878	1.040	0.632	1.710
	People in general thoughts of SH	.176	0.763	0.516	1.129
	People in general SH	.820	1.053	0.677	1.637
	Friends perm of SH	.003	4.363	1.650	11.540
	Parents perm of SH	.865	1.179	0.179	7.779
	Family perm of SH	.749	1.338	0.225	7.940
	Pupils the same age/sex perm of SH	.500	1.466	0.482	4.460
	Pupils at same school perm of SH	.733	1.264	0.329	4.865
	High-school pupils in general perm of SH	.232	0.480	0.145	1.596
	People in general perm of SH	.011	3.494	1.336	9.138
Model 6: Perm of SA X^2 (23) = 203.71, p < .001 R^2 = .47 (Cox & Snell), .63 (Nagelkerke)	Sex (male)	.004	0.345	0.169	0.706
	Age	.160	1.267	0.910	1.764
	Friends thoughts of suicide	.965	1.014	0.555	1.853
	Friends SA	.757	0.875	0.376	2.037
	Parents thoughts of suicide	.999	9.533E9	0.000	-
	Parent SA	.999	0.000	0.000	-
	Family thoughts of suicide	.855	1.065	0.544	2.083
	Family SA	.823	1.213	0.225	6.544
	Pupils the same age/sex thoughts of suicide	.037	1.590	1.029	2.458

Pupils the same age/sex SA	.365	0.836	0.567	1.232
Pupils at same school thoughts of suicide	.979	0.994	0.637	1.552
Pupils at same school SA	.152	1.298	0.909	1.853
High-school pupils in general thoughts of suicide	.322	0.779	0.476	1.277
High-school pupils in general SA	.517	0.873	0.578	1.318
People in general thoughts of suicide	.108	1.395	0.930	2.093
People in general SA	.286	0.816	0.562	1.185
Friends perm of SA	.003	6.208	1.853	20.797
Parents perm of SA	.127	0.195	1.853	1.596
Family perm of SA	.001	29.308	3.895	220.554
Pupils the same age/sex perm of SA	.067	5.806	0.882	38.209
Pupils at same school perm of SA	.999	1.001	0.138	7.257
High-school pupils in general perm of SA	.285	0.358	0.055	2.354
People in general perm of SA	.658	1.222	0.503	2.969

[1] SH = self-harm, [2] SA = suicide attempt, [3] Perm = permissiveness (injunctive norms).

© 2017 by the authors; licensee MDPI, Basel, Switzerland. This article is an open access article distributed under the terms and conditions of the Creative Commons Attribution (CC-BY) license (http://creativecommons.org/licenses/by/4.0/).
